# Cardiovascular health and potential cardiovascular risk factors in young athletes

**DOI:** 10.3389/fcvm.2023.1081675

**Published:** 2023-06-02

**Authors:** Carl Grabitz, Katharina M. Sprung, Laura Amagliani, Nima Memaran, Bernhard M. W. Schmidt, Uwe Tegtbur, Jeannine von der Born, Arno Kerling, Anette Melk

**Affiliations:** ^1^ Department of Pediatric Kidney, Liver and Metabolic Diseases, Hannover Medical School, Hannover, Germany; ^2^ Institute of Sports Medicine, Hannover Medical School, Hannover, Germany; ^3^ Department of Nephrology and Hypertension, Hannover Medical School, Hannover, Germany

**Keywords:** child, adolescent, pulse wave velocity, left ventricular mass, blood pressure

## Abstract

**Introduction:**

Cardiovascular disease remains the most common cause of death worldwide, and early manifestations are increasingly identified in childhood and adolescence. With physical inactivity being the most prevalent modifiable risk factor, the risk for cardiovascular disease is deemed low in people engaging in regular physical exercise. The aim of this study was to investigate early markers and drivers of cardiovascular disease in young athletes pursuing a career in competitive sports.

**Methods:**

One hundred and five athletes (65 males, mean age 15.7 ± 3.7 years) were characterized by measurement of body impedance to estimate body fat, blood pressure (BP), carotid femoral pulse wave velocity (PWV) to evaluate arterial elasticity, ergometry to assess peak power output, echocardiography to calculate left ventricular mass, and blood tests.

**Results:**

Systolic BP was elevated in 12.6% and thereby more than twice as high as expected for the normal population. Similarly, structural vascular and cardiac changes represented by elevated PWV and left ventricular mass were found in 9.5% and 10.3%. Higher PWV was independently associated with higher systolic BP (*β *= 0.0186, *p* < 0.0001), which in turn was closely correlated to hemoglobin levels (*β *= 0.1252, *p* = 0.0435). In this population, increased left ventricular mass was associated with lower resting heart rate (*β *= −0.5187, *p* = 0.0052), higher metabolic equivalent hours (*β *= 0.1303, *p* = 0.0002), sport disciplines with high dynamic component (*β *= 17.45, *p* = 0.0009), and also higher systolic BP (*β *= 0.4715, *p* = 0.0354).

**Conclusion:**

Despite regular physical exercise and in the absence of obesity, we found an unexpected high rate of cardiovascular risk factors. The association of PWV, systolic BP, and hemoglobin suggested a possible link between training-induced raised hemoglobin levels and altered vascular properties. Our results point toward the need for thorough medical examinations in this seemingly healthy cohort of children and young adults. Long-term follow-up of individuals who started excessive physical exercise at a young age seems warranted to further explore the potential adverse effects on vascular health.

## Introduction

Cardiovascular (CV) disease is a leading cause of death and disability worldwide (1). The World Health Organization (WHO) accounts 32% of all deaths (17.9 million per year) mainly to myocardial infarction and stroke (https://www.who.int/health-topics/cardiovascular-disease). Physical inactivity is the most prevalent modifiable risk factor in this regard (2) prompting the WHO to devise a global action plan (3).

While clinical manifest CV disease usually presents in adulthood, the underling arteriosclerotic process begins much earlier (4). Starting already in childhood, the fractured elastin lamellae in the aorta and elastic arteries cause continuous remodeling, which leads to vascular stiffening. The lost elasticity raises pulse pressure and the aortic pulse wave velocity (PWV), which can later contribute to the development of left ventricular hypertrophy, cardiac failure, and microvascular disease in highly perfused organs such as the brain and kidneys (4). Although these developments are physiological to a certain extent, the concept of early vascular aging describes an accelerated remodeling process driven by the interaction of genetic predisposition with certain risk factors like arterial hypertension, insulin resistance, microinflammation, and dyslipidemia (5). Especially cardiorespiratory fitness and adiposity are connected to stiffer blood vessels even in young children (6). Symptomatic endpoints such as chronic heart failure, myocardial infarction, and stroke are rare in young individuals. Yet, subclinical changes can be noninvasively assessed by measuring PWV or left ventricular mass (LVM) in children and young adults (7, 8). Both parameters are well-defined surrogate markers and are associated with cardiovascular disease later in life (9, 10).

In this context, it is generally assumed that athletes are in good cardiovascular health; after all the amount of physical activity performed in this group, it must be considered sufficient to comply with the recommendations by the WHO (11). Reports have shown normal to reduced PWV in adult elite athletes performing swimming (12), endurance sports (13), and a diverse set of other sport disciplines (14). However, there is also evidence for strength and resistance training elevating PWV in older adults (15). Indeed, a recent study showed that even in young adults engaging in different sports, there were markedly raised PWV values (16). Increases in LVM, also called *athletes’ heart*, are frequently seen in athletes and are greater in endurance sports (17). This has also been observed in active children and adolescents (18) albeit being not as pronounced (19). Younger athletes appear to have primary chamber dilation and less hypertrophy (20) causing a more eccentric remodeling (21). This hypertrophy is often regarded as a physiological adaption to exercise. Yet in some cases, there is a morphological overlap with primary cardiomyopathies, posing a potential threat to athletes (22). Similar to chances in LVM, there is also mixed evidence for the effect of competitive sports on blood pressure (BP). Up to a third of a large contemporary cohort of young athletes formally fulfilled the criteria of arterial hypertension (23), while others saw a decrease of diastolic BP (24). Of note, young non-endurance athletes in the latter study presented with increased systolic BP. When looking at other known risk factors such as classical blood biomarkers, young athletes tend to have lower levels of low-density lipoprotein and higher levels of high-density lipoprotein (25).

The aim of our study was to investigate the CV risk in young athletes pursuing a career in competitive sports. We comprehensively assessed vascular and cardiac surrogate markers indicative of subclinical CV damage and several blood parameters known to reflect CV risk.

## Methods

This prospective cross-sectional study recruited young athletes from the Olympic Training Centre Lower Saxony in Hannover, Germany, over a period of 4 months (October, November, and December 2016 and September 2017). One hundred and five out of 340 children and young adults between 7.9 and 28.6 years of age, who engaged in their annual sport medical examination, consented to be enrolled. The participants were active in different competitive sports disciplines: tennis (*n* = 7), cycling (*n* = 8), gliding (*n* = 4), basketball (*n* = 19), judo (*n* = 10), karate (*n* = 3), swimming (*n* = 8), field hockey (*n* = 5), rugby (*n* = 7), decathlon (*n* = 1), handball (*n* = 1), sailing (*n* = 12), water ski (*n* = 6), boxing (*n* = 7), and running (*n* = 7). We grouped sport disciplines based on their dynamic or static components, respectively, into high, moderate, and low according to the Mitchell classification (26). The study was approved by the local institutional review board (file number 3339–2016) and, if applicable, the consent from the respective parents was acquired prior to participation.

All examinations were carried out in the morning. The athletes were allowed to have a light breakfast, of their choice, mostly containing long carbohydrates. Examinations always followed the same order: blood samples were taken first, followed by anthropometric measurements, bioimpedance analysis, BP and resting heart rate (HR), ECG, echocardiography, measurement of PWV, and ergometer tests. Bioimpedance analysis was carried out using the InBody720 device (InBody Europe B.V., Eschborn, Germany). Standardized measurement of BP and resting HR were performed in a seated position with a validated oscillometric device (Dinamap, Carescape V100; GE Healthcare, Chicago, IL, United States) after 5 min of rest as described previously (27). Carotid femoral PWV was evaluated using the oscillometric Vicorder device (Skidmore Medical Limited, Bristol, United Kingdom; Software Version 4) according to the recommendations of the Task Force III on clinical applications of arterial stiffness (28).

We calculated *z*-scores for BMI, height, weight (29), body fat percentage (30), BP (31), resting HR (32), and PWV (7). Obesity was defined as BMI *z*-score ≥1.645 (reflecting values above the 95th percentile). Similarly, elevated BP values were defined as either systolic or diastolic BP *z*-scores ≥1.645. In case participants exceeded the upper age range of the respective reference cohort, we calculated their *z*-scores based on the highest age available for reference as the cut-off values generally used in adulthood correspond very well to *z*-scores reflecting the 90th–95th percentiles in adolescents of 16–18 years of age. This approach was taken for BMI, BP, resting HR, as well as body fat and was only used to allow for better visualization of the results.

Transthoracic echocardiography was performed using the ultrasound GE Vivid I, equipped with a 1–5 MHz transducer, according to the recommendations of the American Society of Echocardiography (33). All examinations and measurements were carried out by one experienced consultant specialized in cardiology following a standardized protocol. Left ventricular end-diastolic wall thickness and end-diastolic dimensions were obtained from the parasternal long-axis view at the level of the papillary muscles using M-Mode. Measurements were made only if image quality was excellent and allowed for unequivocal identification of all relevant structures. Relative wall thickness was calculated and a score greater than 0.42 cm was considered abnormal (34). We calculated LVM (35), LVM index (36), and LVM *z*-scores (8).

The athletes’ cardiorespiratory fitness was examined through a slightly modified bicycle ergometry (Viasprint 150P, Ergoline, Bitz, Germany) test (37). In brief, all participants started with a load of 50 W and were increased by 17W every minute (10 W for athletes with a bodyweight below 40 kg). HR was measured continuously; BP and blood lactate concentration (Ebio 6666, Eppendorf, Hamburg) were measured at rest, 1 min after the start of exercise, and every 3 min during the exercise. Termination criterion of the ergometry was the physical exhaustion of the athletes, determined by subjective exhaustion, or if cadence could not be maintained above 60 revelations/min. The maximum wattage was related to body weight and lean body mass.

Questionnaires adapted from the German Health Interview and Examination Survey for Children and Adolescents (38) were used to capture participants’ activities (hours per week spent on training within their respective sport's discipline, other physical activities, and years of involvement in competitive sports). In addition, we retrieved information on pre-existing illnesses of participants. An individual metabolic equivalent (MET) value was assigned to each of the indicated sport disciplines depending on their respective intensity (39). MET hours were then calculated by multiplying the number of training hours (reported by the athletes) with the respective sport-specific MET score.

Blood samples were analyzed in one central laboratory (Klinikum Region Hannover, Hannover, Germany) and included small blood count, electrolytes, creatinine, urea, total bilirubin, lactate dehydrogenase, aspartate transaminase, alanine transaminase, alkaline phosphatase, gamma gamma-glutamyltransferase, creatine kinase, total cholesterol, low-density lipoprotein, high-density lipoprotein, triglycerides, C-reactive protein, and ferritin. The estimated glomerular filtration rate was calculated by the Schwartz bedside formula for athletes who were under 18 years of age (40) and by the CKD-Epi equation for those who were 18 years or older (41). Hemoglobin *z*-scores adjusted for age were calculated using data from the German Health Interview Examination Survey for Children and Adolescents (42).

Statistical analysis was performed using SAS 9.4M6 (Statistical Analysis Software, Cary, NC, United States). Continuous variables are given as mean ± SD. A *p*-value of <0.05 was considered statistically significant. Two-sided *t*-tests were used for the comparison of continuous variables. Data were further evaluated by the use of multivariable regression models. For the outcomes, LVM and PWV, we started with setting up a basic model corrected for the covariates sex, age, and height and using raw data (not *z*-scores) as outcome. Potential predictors were chosen based on prior knowledge: systolic BP (7), resting HR (38), sport discipline (26), MET hours (39), and hemoglobin (40). Hemoglobin was also chosen as hemoglobin values were significantly raised in our study group. Each of these predictors was added separately as an independent variable to the basic models. For the full models, only predictors showing a *p*-value <0.05 were selected.

## Results

**Figure 1:**
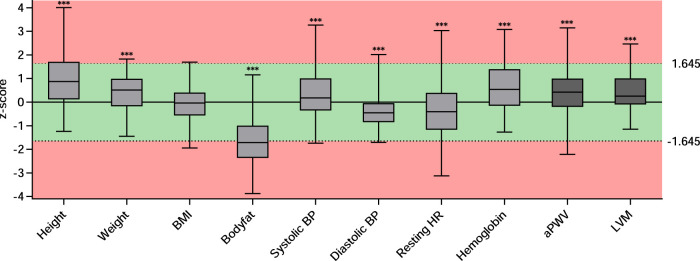
*z*-scores for parameters resembling cardiovascular risk or being indicative for cardiovascular health. Data are presented as boxplots of the available *z*-scores. The box includes the interquartile range with the median denoted in the middle. Minimum and maximum are depicted as whiskers. *** indicates a significance of *p* < 0.001 in a one sample *t*-test compared to “0.” The red background represents *z*-scores smaller than −1.645 or greater than 1.645 corresponding to the 5th and 95th percentile, respectively. BMI, body mass index; BP, blood pressure; HR, heart rate; PWV, pulse wave velocity; LVM, left ventricular mass.

We enrolled a total of 105 young athletes (65 males; 62%) at the Olympic Training Centre Lower Saxony in Hannover. Table 1 shows the basic characteristics of all participants, also categorized by sex. The mean age was 15.7 ± 3.7 (range 7–28) years. As reflected by the calculated *z*-scores (Figure 1), athletes were significantly taller and heavier compared to the WHO reference cohort (*p* < 0.0001 for height and weight), while their BMI was close to average. Athletes’ body composition showed a significantly lower proportion of fat (*p* < 0.0001 compared to the underlying reference cohort; Figure 1). The athletes participated in competitive sports for an average of 6.5 ± 3.3 years and were currently attending training sessions for 9.4 ± 5.4 h per week resulting in an average of 96.3 ± 71.3 MET hours. The overall relative maximum power output was 3.9 ± 0.5 W/kg. Compared to a reference cohort consisting of healthy children, the participants of our study were in the upper average range (43). None of the participants reported smoking.

**Table 1:** Basic characteristics of all participants and according to sex.

	Total (*n* = 105)	Boys (*n* = 65)	Girls (*n* = 40)	
Body composition	M ± SD	M ± SD	M ± SD	*p*
Age, years	15.7 ± 3.7	16 ± 3.8	15.1 ± 3.4	0.2203
Height, cm	170.2 ± 12.2	172.8 ± 12.2	166 ± 10.9	**0**.**0045**
Weight, kg	59.4 ± 14.6	61.7 ± 16.3	55.8 ± 10.6	**0**.**0441**
BMI, kg/m²	20.2 ± 3.1	20.3 ± 3.4	20.1 ± 2.4	0.7337
Body fat, %	12.3 ± 6.5	9.7 ± 5	16.5 ± 6.4	**<**.**0001**
Muscle mass, %	48.8 ± 3.9	50.4 ± 3.1	46.1 ± 3.7	**0**.**0004**
Lean body mass, kg	45.0 ± 12.4	48.5 ± 13.6	38.9 ± 6.8	**0**.**0002**
Training	M ± SD	M ± SD	M ± SD	
Experience, years of involvement	6.5 ± 3.3	6.1 ± 3.4	7.1 ± 3.1	0.1573
Current exposure, hours of training/week	9.4 ± 5.4	9.3 ± 5	9.5 ± 6.1	0.8558
Intensity, MET hours	96.3 ± 71.3	94 ± 67.3	99.8 ± 77.3	0.6943
Max. power output, W	229.2 ± 59.1	243.5 ± 62.8	206 ± 44.3	**0**.**0015**
Max. power output, watt/kg bodyweight	3.9 ± 0.5	4.0 ± 0.5	3.7 ± 0.5	**0**.**0079**
Max. power output, watt/kg lean body mass	4.4 ± 0.5	4.4 ± 0.5	4.5 ± 0.6	0.7375

*p-*value of <0.05 in bold. M, mean; SD, standard derivation; BMI, body mass index; MET, metabolic equivalent of task; *p*, *p-*value.

Table 2 gives an overview of additional parameters assessed to determine cardiovascular health and blood parameters resembling classical and nonclassical cardiovascular risk factors. Mean systolic BP was 118 ± 13 mmHg and mean diastolic BP was 62 ± 7 mmHg. Athletes displayed higher mean systolic BP (*z*-score 0.4 ± 1) and lower mean diastolic BP value (*z*-score −0.4 ± 0.6) compared to reference values (*p* = 0.0006 and *p* < 0.0001, respectively, Figure 1). Twelve athletes (12.6%) had elevated systolic BP levels; only one athlete (1.05%) displayed an elevated diastolic BP. Mean resting HR was 71.2 ± 13.7 and was significantly lower than reference (*z*-score −0.4 ± 1.4; *p* = 0.00015, Figure 1).

**Table 2:** Parameters either resembling classical and nonclassical cardiovascular risk factors or being indicative of cardiovascular health.

	Total (*n *= 105)	Boys (*n* = 65)	Girls (*n* = 40)	
Cardiovascular parameters	M ± SD	M ± SD	M ± SD	*p*
Systolic BP, mmHg	117.6 ± 12.6	119.5 ± 12.8	114.5 ± 11.9	**0**.**0479**
Diastolic BP, mmHg	61.6 ± 7.1	61.4 ± 7.4	62 ± 6.8	0.7110
Resting heart rate, bpm	71.2 ± 13.7	70.5 ± 13.8	72.2 ± 13.7	0.5514
PWV, m/s	5.6 ± 0.6	5.6 ± 0.6	5.4 ± 0.6	0.0840
Left ventricular mass, g	132.4 ± 40	146.4 ± 40.5	113.4 ± 30.6	**<**.**0001**
Left ventricular mass index, g/m^2.16^	40.1 ± 8.8	42.7 ± 8.9	36.7 ± 7.5	**0**.**0013**
Left ventricle relative wall thickness, cm	0.3 ± 0.03	0.3 ± 0.03	0.3 ± 0.03	0.9247
Laboratory tests	M ± SD	M ± SD	M ± SD	
Hemoglobin, g/dl	14.3 ± 1	14.6 ± 1.1	13.9 ± 0.8	**0**.**0003**
Cholesterol, mg/dl	158.5 ± 30.5	155.4 ± 32.6	163.6 ± 26.3	0.2128
LDL, mg/L	96.8 ± 22.5	95.9 ± 24.5	98.1 ± 19	0.6481
HDL, mg/dl	54.9 ± 11.9	53.1 ± 11.1	58.1 ± 12.7	**0**.**0486**
Creatinine, mg/dl	0.75 ± 0.15	0.8 ± 0.2	0.7 ± 0.1	0.1174
Estimated GFR, ml/min/1.73 m^2^	102.3 ± 15.1	104.4 ± 15.9	98.7 ± 13	0.0680
Bilirubin, mg/dl	0.7 ± 0.4	0.6 ± 0.3	0.7 ± 0.4	0.7565
AST, U/L	26.4 ± 9.2	28.1 ± 10.5	23.7 ± 5.7	**0**.**0283**
ALT, U/L	18.7 ± 7.7	20.7 ± 8.5	15.3 ± 4.8	**0**.**0009**
Alkaline phosphatase, U/L	204.4 ± 134.6	231.2 ± 152.3	160 ± 82.8	**0**.**0126**
LDH, U/L	182.3 ± 36.7	186.8 ± 37.3	174.9 ± 35.1	0.1311
CK, U/L	182.2 ± 158.7	202.1 ± 179.4	148.1 ± 109.3	0.1006

*p-*value. *p*-value of <0.05 in bold. M, mean; SD, standard derivation; BP, blood pressure; LDL, low-density lipoprotein; HDL, high-density lipoprotein; GFR, glomerular filtration rate; AST, aspartate-aminotransferase; ALT, alanine-aminotransferase; LDH, lactate dehydrogenase; CK, creatine kinase; PWV, pulse wave velocity; *p*.

Laboratory parameters demonstrated normal kidney and liver function, no overt dyslipidemia, and no sign of myolysis or anemia. The athletes had higher levels of hemoglobin with a *z*-score of 0.6 ± 1.1 compared to the KIGGS-reference cohort (*p* < 0.0001, Figure 1). Of note, boys demonstrated a lower hemoglobin *z*-score when compared to girls (see Supplementary Material Table S1 and Figure S1).

In addition to risk factors, we assessed structural vascular and cardiac changes indicative for cardiovascular end organ damage (Table 2). Mean PWV was 5.6 ± 0.6 m/s. The average *z*-score of 0.4 ± 0.9 was elevated and significantly higher than in the underlying reference cohort (*p* < 0.0001, Figure 1). Nine athletes (9.45%) displayed elevated PWV values. Regression models showed that greater age and height were independent determinants of higher PWV [(a) in Table 3]. When added individually to the basic model for PWV, higher systolic BP (*p* < 0.0001), more MET hours (*p* = 0.0182), and higher hemoglobin (*p* = 0.0435) had a significant positive effect on PWV, while resting HR and sport discipline did not independently influence PWV [(a) in Table 4]. In the full model [(b) in Table 4], systolic BP and MET hours were significantly positive related to PWV.

**Table 3:** Basic models for (a) PWV and (b) LVM corrected for sex, age, and height.

Variables	(a) PWV (*R*^2 ^= 0.3226)			(b) LVM (*R*^2 ^= 0.6549)		
	*β*	SE	*p*	*β*	SE	*p*
Intercept	1.4932	0.8336	**0.0766**	−181.76	40.428	**<.0001**
Female (ref: male)	−0.0174	0.1166	0.8818	−13.368	5.5158	**0**.**0175**
Age	0.0403	0.0167	**0**.**0181**	3.9496	0.8414	**<**.**0001**
Height	0.0202	0.0054	**0**.**0003**	1.5034	0.2661	**<**.**0001**

*p*-value of <0.05 in bold. PWV, pulse wave velocity; LVM, left ventricular mass; β, regression coefficient; SE, standard error; *R*^2^, explained variance.

**Table 4:** Advanced models for PWV: (a) basic model including only one additional independent variable at a time; (b) full model with all variables.

Variables	a: Basic model + 1			b: Full model (*R*^2 ^= 0.4256)		
	*β*	SE	*p*	*β*	SE	*P*
Intercept	*For details please refer to supplement table S4a–S9a*			0.7735	1.0107	0.4463
Female (ref: male)				0.0154	0.1125	0.8917
Age				0.0115	0.0173	0.5063
Height				0.0111	0.0055	**0**.**0455**
Systolic BP mmHg	0.0186	0.0043	**<**.**0001**	0.0160	0.0045	**0**.**0005**
Resting HR bpm	−0.0018	0.0041	0.6572		−	** **
MET hours	0.0019	0.0008	**0**.**0182**	0.0016	0.0008	**0**.**0394**
Hemoglobin, g/dl	0.1252	0.0611	**0**.**0435**	0.0457	0.0587	0.4393
High dynamic component (ref.: low and moderate)	0.0921	0.1158	0.4285		−	

*p*-value of <0.05 in bold. PWV, pulse wave velocity; β, regression coefficient; SE, standard error; BP, blood pressure, HR, heart rate; MET, metabolic equivalent of task; *R*^2^, explained variance.

Mean LVM was 132.4 ± 40 g and LVM *z*-score was higher in our cohort than in the reference cohort (8) (*z*-score 0.4 ± 1, *p* < 0.0001; Figure 1) with elevated LVM in nine athletes (10.3%). When the different sport disciplines were classified based on their dynamic component, athletes engaging in sports with a high dynamic component displayed significantly higher LVM (Supplementary Material Table S2). There was no difference in LVM according to the static component (Supplementary Material Table S3). Regression models showed greater age, height, and male sex were significantly associated with higher LVM [(b) in Table 3]. Adding systolic BP (*p* = 0.0354), MET hours (*p* = 0.0002), and high dynamic component of sports discipline (*p* = 0.0009) individually to the basic model for LVM turned out to have a significant positive effect on LVM, while resting HR demonstrated an inverse effect (*p* = 0.0052). Hemoglobin did not independently influence LVM (Table 5). In the full model, systolic BP, resting heart rate, and high dynamic component of sports discipline retained their significant independent effect on LVM [(b) in Table 5].

**Table 5:** Advanced models for LVM: (a) basic model including only one additional independent variable at a time; (b) full model with all variables.

Variables	a: Basic model + 1			b: Full model (*R*^2 ^= 0. 7677)		
	*β*	SE	*p*	*β*	SE	*P*
Intercept	*For details please refer to supplement table S4b–S9b*			−148.04	41.483	**0**.**0006**
Female (ref: male)				−12.282	4.6259	**0**.**0097**
Age				3.7895	0.7845	**<**.**0001**
Height				1.0903	0.2450	**<**.**0001**
Systolic BP, mmHg	0.4715	0.2204	**0**.**0354**	0.5402	0.1911	**0**.**0061**
Resting HR, bpm	−0.5187	0.1805	**0**.**0052**	−0.5191	0.1648	**0**.**0024**
MET hours	0.1303	0.0339	**0**.**0002**	0.0536	0.0374	0.1560
Hemoglobin, g/dl	−1.9466	3.0329	0.5228		−	
High dynamic component (ref.: low and moderate)	17.450	5.0435	**0**.**0009**	12.315	5.3338	**0**.**0237**

*p*-value of <0.05 in bold. LVM, left ventricular mass; β, regression coefficient; SE, standard error; BP, blood pressure, HR, heart rate; MET, metabolic equivalent of task; *R*^2^, explained variance.

## Discussion

This observational study characterized a cohort of young athletes pursuing a career in competitive sports. In this seemingly healthy population on regular exercise and without obesity, we found a high rate of potential cardiovascular risk factors, illustrating the importance of regular follow-up by sports medicine. Systolic BP elevation in more than 10% of the participants reflects a rate twice as high as to what one expects in the normal population. Similarly, structural vascular and cardiac changes represented by elevated PWV and LVM were found twice more often. Higher PWV was independently associated with higher systolic BP, which in turn was closely correlated with higher hemoglobin levels. This suggested a possible link between training-induced increased hemoglobin levels and vascular stiffness in a small subgroup of this population. Increased left ventricular mass, often discussed as a physiological reaction to physical exercise and higher fitness level, was clearly associated with lower resting HR and high dynamic component of sport discipline, but also higher systolic BP in our cohort.

Our study extends previous findings, which showed elevated PWV levels in young athletes (16), by providing additional data that advance our understanding of potential drivers of accelerated PWV. The full multivariable model confirms that BP significantly enhances PWV, which has already been demonstrated in healthy populations (7) and several patient groups (44). The effect of systolic BP superseded other independent variables, like hemoglobin, which we had found of importance when added to the basic model. Interestingly, Chen et al. observed a correlation of higher hemoglobin level with BP in 3,776 healthy children (45). They hypothesized that hemoglobin in higher concentrations acts as a nitric oxide (NO) scavenger and leads to less free NO causing vasoconstriction. This vasoconstriction could then cause increased systolic BP and over a longer time period may also explain our observation of faster PWV. Thereby, BP and hemoglobin would be interdependent parts in a common chain of effects; hence, after adding both into one model, hemoglobin is no longer independently associated with PWV. Along those lines, a positive correlation between hemoglobin and increased PWV was previously described by others in middle-aged adults (46) and elderly women (47). These findings have to be seen in context with the markedly increased hemoglobin *z*-scores in our cohort. However, physical exercise has been widely described to enhance NO release and thereby to have a positive effect on vascular function in athletes (48).

Of note, 12% of the characterized athletes had an elevated systolic along with low-normal diastolic BP. Whether this condition reflects an innocent, “spurious” phenotype or whether it is a true form of hypertension requiring follow-up and even treatment is heavily debated (49). While a large study found middle-aged adults with isolated systolic hypertension to have a higher risk for cardiovascular morbidity and mortality during their 31-year follow up period (50), elevated systolic BP values in young physically active men have long been deemed normal (51). The underlying pathomechanisms differ by age: stiffer vessels due to vascular aging cause both elevated central and brachial BP, whereas in young healthy individuals, central systolic BP might get augmented due to lower HR and higher stroke volume resulting in increased brachial BP (52). The same study found about 20% of young individuals with isolated systolic hypertension, normal stroke volume, and increased PWV, which matches our observations. Isolated systolic hypertension of the young seems to be a heterogeneous condition, and at least in some individuals, it might be associated with premature stiffening of the vasculature and therefore constitutes a true cardiovascular risk. This has recently been highlighted in a study comparing adolescents with isolated systolic hypertension and peers diagnosed with white coat hypertension. The former group demonstrated significantly higher aortic PWV, LVMI, and a higher incidence of left ventricular hypertrophy (53).

Ten percent of the examined athletes demonstrated elevated LVM, for which we demonstrated systolic BP as an associated risk factor on the one hand. On the other hand, we confirmed observations made by others indicating an independent association with both increased MET hours (i.e., increasing intensity and duration of the performed sport) and lower resting HR (54). Moreover, sport disciplines with a high dynamic component were associated with higher LVM. This is in line with observations from a recent large sample of adult athletes (55). As in our full model MET hours did not have an independent significant influence on LVM, the effect of the training intensity might be carried by the highly dynamic component in our cohort. Interestingly, diastolic BP did not independently influence LVM, which supports our finding of elevated systolic blood pressure driving an increase in LVM. As none of the athletes demonstrated an increased relative wall thickness above 0.42 cm, left ventricular hypertrophy can be classified as eccentric (34), which is suggestive for a physiological adaptation to intense exercise. To which extent the described elevation of LVM resembled truly a benign “athletes’ heart” and how it is best differentiated from hypertensive heart disease is subject to ongoing research, in both junior and senior athletes (56, 57). Recently, *z*-scores for LVM adapted to child and adolescent athletes were proposed (58), raising the question whether elevating cut-off levels to have fewer pathological findings is the correct approach in light of the presented data and without any long-term follow-up in these young individuals.

Hemoglobin *z*-score was significantly higher in girls than in boys. A possible explanation for this phenomenon could be amenorrhea in female participants due to a high training load. Amenorrhea is part of “The Female Athletes Triad” (59) as well as osteoporosis and disordered eating, which was not the focus of our study. Still, our data highlight an additional adverse effect on the long-term health of professional female athletes.

Despite our cohort reflecting a broad age range and various sport disciplines, we were able to not only confirm known findings but also discover potential drivers for cardiovascular alterations using multivariable modeling. Like others (16), we sought to categorize sports disciplines into endurance and strength in order to evaluate whether they affect the cardiovascular system differently (60). However, such classification was difficult to apply, since most athletes either engage in disciplines that already feature strength and endurance components like boxing and basketball or follow training schedules that incorporate both components. In addition, our study had a cross-sectional design. These limitations emphasize the need for further investigations with homogenous sport groups or even an interventional and probably longitudinal design. Another limitation is that we did not perform central BP measurements, which would have helped to further assess the young athletes with elevated systolic BP values. We also did not engage in extensive electrophysiological investigations like Holter monitoring. The latter would have uncovered arrhythmias, which are among the leading causes for sudden cardiac death besides congenital heart defects and hypertrophic cardiomyopathies in young athletes (61).

In our cohort of young athletes, we found a high rate of potential cardiovascular risk factors and described an association between increased PWV and systolic BP alongside raised hemoglobin levels. We, like others, assumed an etiological relationship between those parameters linked by NO and therefore identifying hemoglobin as a potential risk factor. This observation is of particular importance since in the context of competitive sports, high normal hemoglobin levels are seen as a marker of good physical capacity because of the strong relationship between total hemoglobin mass and maximal aerobic capacity (62). Taken together, regular sports and exercise prevent risk factors like obesity, dyslipidemia, or hyperglycemia and, therefore, have an overall positive effect on health. Our results point toward the need of regular medical examinations in this seemingly healthy cohort of children and young adults. Long-term follow-up of individuals who started excessive physical exercise at a young age seems warranted to further explore potential adverse effects on vascular health.

## Data Availability

The raw data supporting the conclusions of this article will be made available by the authors, upon reasonable request.
