# Supplementary material for: Cardiovascular health and potential cardiovascular risk factors in young athletes
Source: Front Cardiovasc Med. 2023 Jun 2;10:1081675. doi: 10.3389/fcvm.2023.1081675 (PMC10272594; doi:10.3389/fcvm.2023.1081675)
Supplement: Supplementary file 1 [file Datasheet1.pdf]

Supplementary Material

Cardiovascular health and potential cardiovascular risk factors in young athletes

Carl Grabitz, Katharina M. Sprung, Laura Amagliani, Nima Memaran, Bernhard M.W. Schmidt, Uwe Tegtbur, Jeannine von der Born, Arno Kerling, Anette Melk*

*** Correspondence:** Anette Melk, MD PhD, Melk.Anette@mh-hannover.de

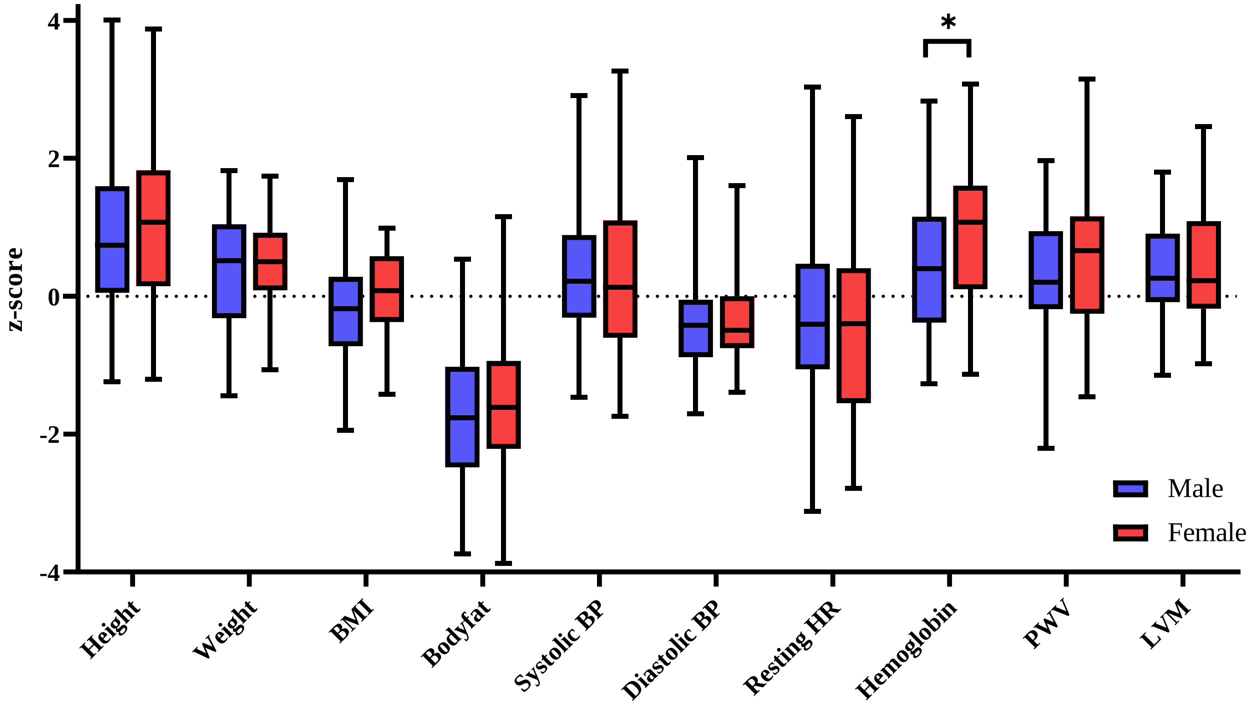

<u>Figure S1:</u> Z-scores for parameters resembling cardiovascular risk or being indicative for cardiovascular health discriminated by sex.

Data are presented as boxplots of the available z-scores. The box includes the interquartile range with the median denoted in the middle. Minimum and maximum are depicted as whiskers. * indicates a significance of $p<0.05$ in a two-sample independent t-test between sexes.

Abbreviations: BMI, body mass index; BP, blood pressure; HR, heart rate; PWV, pulse wave velocity; LVM, left ventricular mass.

Table S1. Z-scores for athletes' parameters.

	**Total (n=105)**	**Boys (n=65)**	**Girls (n=40)**	**p[a] (male vs female)**	**p[b] (vs 0)**
**Variables**	M ± SD	M ± SD	M ± SD		
**Height**	0.9 ± 1.2	0.9 ± 1,2	1 ± 1.3	0.5845	**<.0001**
**Weight**	0.4 ± 0.8	0.4 ± 0.9	0.5 ± 0.7	0.5732	**<.0001**
**BMI**	-0.1 ± 0.7	-0.1 ± 0.8	0 ± 0.6	0.2116	0.3727
**Bodyfat**	-1.6 ± 1.1	-1.7 ± 1.1	-1.5 ± 1	0.366	**<.0001**
**Systolic BP**	0.4 ± 1	0.4 ± 0.9	0.3 ± 1.2	0.7110	**0.0006**
**Diastolic BP**	-0.4 ± 0.6	-0.4 ± 0.6	-0.4 ± 0.6	0.7678	**<.0001**
**Resting HR**	-0.4 ± 1.4	-0.3 ± 1.2	-0.5 ± 1.3	0.5263	**0.00015**
**Hemoglobin**	0.6 ± 1.1	0.5 ± 1	1 ± 1.1	**0.0227**	<.0001
**PWV**	0.4 ± 0.9	0.3 ± 0.8	0.6 ± 1.1	0.2139	**<.0001**
**LVM**	0.4 ± 1.	0.4 ± 0.7	0.5 ± 0.9	0.5723	**<.0001**

Abbreviations: p, p-value; M, mean; SD, standard derivation; BMI, body mass index; BP, blood pressure; HR, heart rate; PWV, pulse wave velocity; LVM, left ventricular mass; PWV, pulse wave velocity; LVM, left ventricular mass
Explanatory notes: [a] two sample unpaired t-test; [b] one sample t-test with 0 as hypothetical mean

Table S2. Athletes' characteristics discriminated by the extent of the dynamic component. The dynamic component of each sport discipline was defined according to the Mitchell classification (26).

	**Low & moderate dynamic component** (n=47)		**High dynamic component** (n=58)		
	**Mean/ frequency**	**SD/%**	**Mean/ frequency**	**SD/%**	**p**
**Females**, percentage	21.0	0.4	19.0	0.3	0.2110
**Age**, years	16.5	4.3	15.0	2.9	0.0366
**Height**, cm	168.1	13.6	172.0	10.6	0.1056
**BMI,** kg/m2	20.9	3.1	19.7	3.0	**0.0386**
**Weight**, kg	60.1	15.4	58.9	14.0	0.6796
**Systolic BP,** mmHg	119.7	11.9	115.9	13.1	0.1251
**Diastolic BP,** mmHg	62.4	7.6	61.0	6.8	0.3121
**Resting heart rate,** bpm	73.4	16.2	69.3	11.1	0.1342
**Estimated GFR,** ml/min/1,73 m^2	93.3	14.1	99.3	17.3	0.0621
**Hemoglobin,** g/dl	14.3	1.0	14.3	1.1	0.9329
**Intensity,** MET-hours	63.5	52.1	120.0	74.2	**<.0001**
**Left ventricular mass,** g	122.9	39.3	143.5	38.2	**0.0152**
**Left ventricular mass index,** $g/m^{2.16}$	38.0	8.3	42.6	8.8	**0.0135**
**PWV,** m/s	5.51	0.69	5.61	0.56	0.4376

For our cohort the low & moderate dynamic component included the following sport disciplines: Judo, karate, sailing, waterski, gliding, rugby, running (sprint). Sport disciplines with a high dynamic component included boxing, decathlon, cycling, basketball, handball, swimming, tennis, running (middle distance)

Table S3. Athletes' characteristics discriminated by the extent of the static component. The static component of each sport discipline was defined according to the Mitchell classification (26).

	**Low & moderate static component** (n=58)		**High static component** (n=47)		
	**Mean/ frequency**	**SD/%**	**Mean/ frequency**	**SD/%**	**p**
**Females**, percentage	21	36%	19	40%	0.6580
**Age**, years	15.2	3.4	16.3	3.9	0.1211
**Height**, cm	172.1	9.6	167.9	14.4	0.0721
**BMI,** kg/m2	19.7	2.8	20.8	3.3	0.0847
**Weight**, kg	59.2	13.1	59.7	16.3	0.8538
**Systolic BP,** mmHg	116.2	12.0	119.3	13.3	0.2164
**Diastolic BP,** mmHg	61.2	6.7	62.2	7.7	0.4785
**Resting heart rate,** bpm	67.1	11.8	76.1	14.4	**0.0006**
**Estimated GFR,** ml/min/1,73 m^2	99.0	17.6	93.8	13.9	0.1077
**Hemoglobin,** g/dl	14.3	1.0	14.4	1.1	0.4063
**Intensity,** MET-hours	98.8	71.1	93.3	72.2	0.7004
**Left ventricular mass,** g	138.5	32.3	127.1	45.1	0.1868
**Left ventricular mass index,** $g/m^{2.16}$	41.1	7.5	39.3	9.8	0.3300
**PWV,** m/s	5.57	0.60	5.55	0.65	0.8338

For our cohort the low & moderate static component included the following sport disciplines: Gliding, rugby, running, basketball, handball, swimming, tennis, field hockey. Sport disciplines with a high static component included judo, karate, sailing, waterski, boxing, decathlon, cycling.

Table S4. Basic models for (a) PWV and (b) LVM corrected for sex, age and height including systolic BP

**Variables**	**a:** PWV			**b:** LVM		
	**β**	**SE**	**p**	**β**	**SE**	**p**
**Intercept**	0.461	0.7999	0.5658	-213.84	42.328	**<.0001**
**Female** (ref: male)	0.0045	0.1068	0.9667	-11.286	5.4876	**0.0429**
**Age**	0.0240	0.0158	0.1327	3.6872	0.8330	**<.0001**
**Height**	0.0149	0.0051	**0.0045**	1.3826	0.2666	**<.0001**
**Systolic BP**	0.0186	0.0043	**<.0001**	0.4715	0.2204	**0.0354**

Abbreviations: PWV, pulse wave velocity; LVM, left ventricular mass; β, regression coefficient; SE, standard error; BP, blood pressure

Table S5. Basic models for (a) PWV and (b) LVM corrected for sex, age and height including diastolic BP

**Variables**	**a:** PWV			**b:** LVM		
	**β**	**SE**	**p**	**β**	**SE**	**p**
**Intercept**	-0.0712	0.9992	0.9434	-211.37	48.703	**<.0001**
**Female** (ref: male)	-0.0675	0.1201	0.5760	-14.660	5.9250	**0.0162**
**Age**	0.0712	0.0371	0.0591	2.0830	2.1925	0.3459
**Height**	0.0160	0.0058	**0.0077**	1.7556	0.3188	**<.0001**
**Diastolic BP**	0.0313	0.0104	**0.0036**	0.2379	0.4115	0.5652

Abbreviations: PWV, pulse wave velocity; LVM, left ventricular mass; β, regression coefficient; SE, standard error; BP, blood pressure

Table S6. Basic models for (a) PWV and (b) LVM corrected for sex, age and height including resting HR

**Variables**	**a:** PWV			**b:** LVM		
	**β**	**SE**	**p**	**β**	**SE**	**p**
**Intercept**	1.7261	0.9872	0.0838	-117.35	44.784	**0.0105**
**Female** (ref: male)	-0.0187	0.1171	0.8738	-13.699	5.2907	**0.0114**
**Age**	0.0399	0.0168	**0.0199**	3.8290	0.8080	**<.0001**
**Height**	0.0197	0.0056	**0.0007**	1.3530	0.2604	**<.0001**
**Resting HR**	-0.0018	0.0041	0.6572	-0.5187	0.1805	**0.0052**

Abbreviations: PWV, pulse wave velocity; LVM, left ventricular mass; β, regression coefficient; SE, standard error; HR, heart rate

Table S7. Basic models for (a) PWV and (b) LVM corrected for sex, age and height including MET-hours

**Variables**	**a:** PWV			**b:** LVM		
	**β**	**SE**	**p**	**β**	**SE**	**p**
**Intercept**	2.0825	0.8701	0.0189	-173.61	39.736	**<.0001**
**Female** (ref: male)	-0.0363	0.1130	0.7486	-15.342	4.9932	**0.0029**
**Age**	0.0281	0.0178	0.1188	3.5896	0.8281	**<.0001**
**Height**	0.0167	0.0055	**0.0034**	1.4170	0.2573	**<.0001**
**MET-hours**	0.0019	0.0008	**0.0182**	0.1303	0.0339	**0.0002**

Abbreviations: PWV, pulse wave velocity; LVM, left ventricular mass; β, regression coefficient; SE, standard error; MET, metabolic equivalent of task

Table S8. Basic models for (a) PWV and (b) LVM corrected for sex, age and height including hemoglobin

**Variables**	**a:** PWV			**b:** LVM		
	**β**	**SE**	**p**	**β**	**SE**	**p**
**Intercept**	0.2274	1.0309	0.8259	-164.92	49.073	**0.0012**
**Female** (ref: male)	0.0503	0.1217	0.6804	-14.193	6.0297	**0.0211**
**Age**	0.0358	0.0168	**0.0355**	4.0605	0.8602	**<.0001**
**Height**	0.0173	0.0056	**0.0025**	1.5620	0.2878	**<.0001**
**Hemoglobin**	0.1252	0.0611	**0.0435**	-1.9466	3.0329	0.5228

Abbreviations: PWV, pulse wave velocity; LVM, left ventricular mass; β, regression coefficient; SE, standard error;

Table S9. Basic models for (a) PWV and (b) LVM corrected for sex, age and height including sport disciplines grouped by dynamic component.

**Variables**	**a:** PWV			**b:** LVM		
	**β**	**SE**	**p**	**β**	**SE**	**p**
**Intercept**	1.5912	0.8443	0.0627	-159.24	38.549	**<.0001**
**Female** (ref: male)	-0.0128	0.1169	0.9134	-13.411	5.1838	**0.0114**
**Age**	0.0450	0.0178	**0.0131**	4.5960	0.8126	**<.0001**
**Height**	0.0189	0.0057	**0.0012**	1.2623	0.2596	**<.0001**
**High dynamic component** (ref low & moderate)	0.0921	0.1158	0.4285	17.450	5.0435	**0.0009**

Abbreviations: PWV, pulse wave velocity; LVM, left ventricular mass; β, regression coefficient; SE, standard error;
